# Sources of information influencing the state-of-the-science gap in hormone replacement therapy usage

**DOI:** 10.1371/journal.pone.0171189

**Published:** 2017-02-03

**Authors:** Fiona Chew, Xianwei Wu

**Affiliations:** 1 S.I. Newhouse School of Public Communications, Syracuse University, Syracuse, New York, United States of America; 2 School of Journalism and Mass Communication, The University of Iowa, Iowa City, Iowa, United States of America; Universidad de las Palmas de Gran Canaria, SPAIN

## Abstract

**Objective:**

Medical reviews and research comprise a key information source for news media stories on medical therapies and innovations as well as for physicians in updating their practice. The present study examined medical review journal articles, physician surveys and news media coverage of hormone replacement therapy (HT) to assess the relationship between the three information sources and whether/if they contributed to a state-of-the-science gap (a condition when the evaluation of a medical condition or therapy ascertained by the highest standards of investigation is incongruent with the science-in-practice such as physician recommendations and patient actions).

**Methods:**

We content-analyzed 177 randomly sampled HT medical reviews between 2002 and 2014, and HT news valence in three major TV networks, newspapers and magazines/internet sites in 2002–2003, 2008–2009 and 2012–14. The focus in both analyses was whether HT benefits outweighed risks, risks outweighed benefits or both risks and benefits were presented. We also qualitatively content-analyzed all 19 surveys of US physicians’ HT recommendations from 2002 to 2009, and 2012 to 2014.

**Results:**

Medical reviews yielded a mixed picture about HT (40.1% benefits, 26.0% risks, and 33.9% both benefits and risks). While a majority of physician surveys were pro-HT 10/19), eight showed varied attitudes and one was negative. Newspaper and television coverage reflected a pro and con balance while magazine stories were more positive in the later reporting period.

**Conclusion:**

Medical journal review articles, physicians, and media reports all provide varying view points towards hormone therapy use thus leading to limited knowledge about the actual risks and benefits of HT among peri- and menopausal women and a state-of-the-science gap.

## Introduction

A growing number of recent reports contends that the benefits of hormone replacement therapy (HT) can be optimized and risks mitigated for newly menopausal [1–5] and postmenopausal women [6,7]. In contrast, the US Preventive Services Task Force recommends against the use of HT for the prevention of chronic conditions in postmenopausal women because the risks outweigh the benefits [8]. The recommendation was predicated on the findings of the Women’s Health Initiative (WHI), a large-scale, longitudinal, randomized, controlled trial involving 16,608 women aged 50 to 79 which established that HT use increased the risks of breast cancer, heart attack, stroke, and blood clots [9,10]. Later analyses of the WHI data further differentiated effects by age of initiation, time from menopause and HT used (estrogen with progestin or estrogen alone) [11,12]. These studies reported that menopausal HT use was appropriate for vasomotor symptom management but not for the prevention of coronary heart disease [9–12]. To better understand the gap between favorable and unfavorable HT recommendations, this study examined key sources of information about HT to assess their valence and influence, and how these may contribute to the state-of-the-science gap.

In America’s youth-oriented culture, menopause, a natural stage in aging, has been considered a condition to be postponed or prevented with the use of hormone replacement therapy [13]. Popularized by New York gynecologist, Robert Wilson’s 1966 book *Feminine Forever*, HT was regularly prescribed to control menopausal symptoms and prolong ovulation and a youthful appearance [14]. It was a little-known fact that Wilson’s work publicizing the benefits of HT was funded by hormone manufacturers, Searle, Upjohn and Ayerst Laboratories [15]. In 2002, when findings of the Women’s Health Initiative (WHI) study [9,10] were published, HT use dramatically declined [16–18].

Prior to this date, in spite of growing evidence from epidemiological studies demonstrating the HT-breast cancer link, HT prescriptions peaked at 92 million in 2000, then fell to 56.9 million in 2003 after the WHI results were made known [19]. In order to encourage sales, Wyeth (now part of Pfizer in 2009), the maker of Prempro, Premarin and Premphase developed a medical marketing strategy of publishing in the medical publications via various proxies facilitated through a medical education and communications company, DesignWrite [20–22]. The rationale for authoring medical journal review articles was based on research showing that physicians relied heavily on “journal articles for credible product information.” [20] (p. 3). In addition, the use of medical journals by physicians for updating medical knowledge is well documented [23–26].

The medical marketing strategy consisted of authoring review manuscripts that emphasized HT benefits over risks, identifying prominent experts and offering them study authorship [20–22]. Between 1998 and 2005, over 50 such manuscripts were published [20–22]. The result of Wyeth’s strategy is evident in a recent study that demonstrated promotional tones in selected medical journal articles about hormone therapy and pointed to a link between industry funding and drug promotion in published journal articles [27].

In 2003, Wyeth received FDA approval for low dosage HT and launched medical marketing efforts to position low dosage HT as safe and efficacious [21–23]. Between 2003 and 2009, prescriptions for low dose HT increased [8] in spite of the lack of evidence that low dose HT is safe [28].

An estimated 6,000 women reach menopause in the U.S. every day, which amounts to over 2 million American women per year [29], and many seek therapies to relieve menopause symptoms (such as hot flashes, night sweats and vaginal dryness). Are they able to access comprehensive information to facilitate optimal decision-making regarding appropriate therapies, particularly HT? The present study seeks to address whether medical marketing of HT through medical journal review articles contributed to a state-of-the-science gap in HT usage. This gap is conceptualized as a difference between what is known about the science of HT by scientists and what is practical knowledge and application by health professionals and the general public.

Specifically, the study examines medical review journal articles, surveys of physician and news media coverage of HT to assess the content and relationship between these three information sources. For mass media coverage of hormone therapy, the present study focuses on TV, newspapers and magazines/internet sites as these are the means by which health information diffuses to the public. It proposes to identify key sources of HT information and information paths that lead to HT use.

### State-of-the-science gap

The state-of-the-science gap describes a condition when the state-of-the-science—the assessment of a medical condition ascertained by the highest standards of scientific investigation—is incongruent with the science-in-practice [30]. The state-of-the-science regarding medical therapies, procedures and technology in the U.S. is typically determined by the National Institutes of Health (NIH) through the Consensus Conference during which an independent expert panel assesses the evidence pertaining to the medical condition. It then issues a statement about the medical condition that provides the basis for policy decisions and health guidelines in the country [31]. Science-in-practice refers to physicians’ practice and recommendations, news media reports, and public understanding and behavior.

A previous study identified a state-of-the-science gap in mammography guidelines for women aged 40 –as recommended by the American College of Radiology, women begin their baseline mammography at age 40, a practice that is not supported by the scientific evidence [30]. It attributed the gap to two factors: 1) advocacy by multiple interest groups with goals ranging from providing insurance coverage for the widest reach of women, to increasing revenues for the medical-technology and related industries, and the 2) magazine publication pressures of providing actionable or service information in a sea of controversy [30].

In the present study, we content-analyzed a random sample of medical journal HT review articles and all surveys of US physicians’ HT attitudes and recommendations. We then randomly sampled and content analyzed the amount of news media coverage of HT in major television news media, newspapers and magazines including their internet sites. One of our goals was to understand the conditions of information discrepancy because a gap in knowledge and practice may lead to a deficient understanding of medical science and its opportunities, poor decision making in quality-of-health issues, health disparities and a diminished quality of life.

#### The State-of-the-science in HT usage

The WHI is considered a landmark study conclusively demonstrating the link between the most commonly used combined hormone preparation of estrogen plus progestin and adverse outcomes such as breast cancer and CVD [9,10]. It comprised an NIH randomized, controlled trial (RCT) enrolling 16,608 female subjects aged 50–79 who were randomly assigned to either a group receiving HT (conjugated equine estrogens) or a placebo. Beginning in 1997, the planned 8.5 years study duration was cut short in 2002 when results showed that health risks outweighed benefits in the HT group. These included CHD, invasive breast cancer, stroke, and pulmonary embolism [9,10].

The 32-page NIH Consensus Statement on “Management of menopause-related symptoms” issued in 2005 references the WHI for identifying important risk factors associated with the use of HT. It goes on to caution women seeking HT to assess the risks and benefits with their physicians as decision-making about HT use requires “personal knowledge and balancing of these risks.” [31]. The statement suggests that other potential alternatives to estrogen are present and that their effectiveness and long-term safety require further study [32].

Currently, the Federal Drug Administration (FDA) states that there is no knowledge about whether two low-dose HT drugs, Premarin and Prempro, which the regulatory agency approved in 2003, are safer in low doses [28]. Additionally, it cannot make recommendations until studies are conducted [32]. It does state that generally, “medicines should be used at the lowest effective dose” [32].

### HT usage among women post WHI

Post WHI, many women stopped using and were more reluctant to continue using HT. Five national US surveys with sample sizes in excess of 500 participants reported the following trends. A 2002 survey of 757 recent HT users found that a large majority (81%) of the patients reported changing their HT usage, including 63% who discontinued using HT after learning about the WHI findings [17]. In another study, a majority (64%) of 819 women aged 40 to 79 heard about the groundbreaking study, nearly 6 in 10 (57%) worried about the study outcomes and three—quarters expressed confusion about HT use [33]. A slight majority (56%) of 670 HT users aged 50 to 69 elected to stop using the drug after WHI was published [16], while nearly a third (31.8%) of 1,659 women aged 50+ in a 2004 survey stopped using HT [34]. A longitudinal survey of 3,853 women aged 50+ found that HT use declined from 57% in 2001 to 28% in 2002 to 12% in 2004 [35].

#### Women’s knowledge of HT and reliance on physicians

In general, research showed that women had a low awareness of the risks and benefits of HT even after the publication of WHI and relied on their physicians for HT information. A 2004 study of 781 women found that fewer than a third (29%) was aware of the WHI findings [36]. Additionally, these women were more likely to have talked with their physicians which suggests that talking to physicians may have led to higher HT knowledge. Post WHI, physicians were the number one source for HT issues. One study showed that of 689 women surveyed, a majority (79.7%) viewed physicians as an important information source for menopausal information [37]. In addition, physicians also enjoyed the highest mean ratings for trustworthiness, knowledgeability and helpfulness among women in all stages of menopause [37]. These studies illustrate that physicians comprised the single most important resource for women when they are making decisions about HT usage during menopause.

### Media impact on HT

On the topic of prescriptions drugs, most Americans (85%) find that their health professionals, such as doctors, provide the most helpful information [38]. Since they tend to see their doctors about three times a year [39,40], they may not access this source frequently. Instead they may more likely turn to mediated sources of information. Television is the key source of health information followed by books, magazines and newspapers [41,42]. Internet and print materials also constitute primary sources of information on health and medical issues [43].

Magazines specifically provide informative health information as readers appear to be more knowledgeable about health matters than non-magazine readers [44]. Magazines were also reported as the most trusted media source when it comes to health information [45,46]. In particular, magazines are more likely to favor practical knowledge, helpful tips and advice as part of their service philosophy [30].

Besides traditional media, the internet has also become one the most important channels for general health information. A Health Information National Trends Survey indicated that 63.5% of the online population reported going online for health information in the past 12 months, and 48.6% reported going online for health information first vs. 10.9% who went to their physicians first [38]. Also, people who use the internet for health information have generally higher health consciousness and are more health information oriented [47]. The ways in which these various media, particularly television, newspapers, magazines and the internet cover the HT debate may also affect the state-of-the-science gap.

In studies reporting women’s sources of information for learning about HT, media comprise important sources, although lower in frequency of use than physicians. In a survey of 689 women, 55.7% cited print media (no differentiation between magazines and newspapers) as a source of information while the internet was cited by 29.2% as an information source [48].

Other media focused studies showed that 72% of 97 women reported that physicians are the main influence on their HT usage and 10% mentioned medical reports [49]. Results from the WHI study influenced 53% of the women to change their HT usage pattern: either stop, decrease use, stop and restart or plan to stop [46].

Medical researchers studied coverage of HT before and after the release of WHI findings in six local or national newspapers. They found that coverage of HT increased significantly in June, 2002 due to the publication of WHI (from 11 per month in May to 197 per month in June) [50]. The study also found that reports became more negative (more reports of risks than benefits) in the two months prior to the WHI publication and after it. But at the same time, HT risks and benefits were more clearly explained after June 2002. Coverage providing explanations ranged from 12.7% in 2000 to 34% in July, 2002 to 38.2% in October, 2003 [50].

In 2007, these researchers conducted a follow-up study examining exposure to media reports of HT harms [51]. They content analyzed 22 newspapers in seven geographic areas published from July to October, 2002, and calculated average distribution for each area according to circulation. The study also sampled 327,144 postmenopausal women from July, 2001 to December, 2002 at seven sites, about their HT use for comparison purposes. Results showed that while a majority of women were only exposed to one article about the harmful effects of HT by July 2002, there was a directional relationship between the level of exposure to negative HT reports and the level of HT cessation. Among the HT users, 27% were exposed to three or more negative articles of HT, while 31% were only exposed to less than one article [51]. This was reinforced in a post WHI survey among a subgroup of 175 women aged 45 to 61 who discontinued HT use—41.4% did so based on news reports compared to 19.7% whose physicians recommended cessation [51]. In addition, a 2013 study of 515 women with menopausal symptoms who discussed symptom management with their health providers demonstrated a “considerable lack of knowledge about these symptoms and HT risks” [52], suggesting many women do not receive enough information from their health providers. All in all, studies demonstrated that news media are effective information sources for women’s decision making process, especially with repeated exposures.

### HT prescription volume after the WHI

Prescription data showed declines in 2002 [18]. The National Prescription Audit (NPA) database and National Disease and Therapeutic Index (NDTI) which tracks the number of HT prescriptions written during patient visits to physicians’ office from January 2001 to June, 2003 showed that immediately after WHI’s publication, prescriptions for Prempro (oral EPT) decreased 66% and Premarin (oral ET) decreased 33% [18]. Long-term prescription data from May, 1998 to May, 2003 using Medco Health Database similarly showed that within three months of WHI’s publication, the monthly prescription rates of HT declined from 12.5% to 9.4%, and HT cessation among users significantly increased from 53% in 2001 to 67% in 2002 [53]. A separate three-year study confirmed this trend [54]. Using the National Ambulatory Medical Care Survey (NAMCS) and the National Hospital Ambulatory Medical Care Survey (NHAMCS) provided by the CDC which tracks outpatient visits to non-federal physicians’ offices and hospitals, results showed that HT prescription decreased from 26.5 million in 2001 to 16.9 million in 2003, and the drop for EPT prescription was more significant than ET prescriptions (44% vs. 35% decrease) [54].

### Hypotheses and questions

The following hypotheses and questions guided the present study:

Q1: Post WHI, what are medical review articles’ HT orientation and how will they contribute to a state-of-the-science gap?

Q2: Post WHI, what are physicians’ HT attitudes and how will they contribute to the state-of-the-science?

H1A: Post WHI, media coverage of HT by television news media will reflect coverage consistent with the state-of-the-science that HT risks outweigh the benefits.

H1B: Post WHI, media coverage of HT by newspaper outlets will reflect coverage consistent with the state-of-the-science that HT risks outweigh the benefits.

H1C: Post WHI, in line with providing service information, women’s magazines and their internet coverage of HT will favor HT use and comprise a state-of-the-science gap.

## Materials and methods

To address the above hypotheses, we used content analysis in our study. First, we content-analyzed a random sample of post WHI HT medical review articles from 2002 to 2014. Next we qualitatively content-analyzed all HT attitude surveys of US physicians from 2002 to 2009, and from 2012 to 2014 after the US Preventive Services Task Force (USPSTF) issued its report in October 2012 reaffirming the WHI recommendations [55]. Both types of articles were published in peer reviewed journals. Finally, we content analyzed all HT usage coverage by three television networks, three newspapers and three magazines (which targeted a sizeable proportion of the menopausal woman aged 50 to 60) for three key periods, 2002–2003 (just after the WHI results), 2008–2009 (five to six years after the WHI results) and 2012–2014 (after the USPSTF report’s release).

### Data sources and searches

#### Medical review journal articles analysis

Since Wyeth’s medical marketing strategy focused primarily on authoring and publishing HT review articles, we randomly sampled HT medical review journal articles written after WHI. We used the search terms, “hormone replacement therapy” or “menopausal hormone therapy” within the title and abstracts, and limited the results to English language review articles published after July 2002 to December 2014. The search returned 1,284 articles, which comprised our population frame from which we selected a sample of 200 articles through random number assignment. After removing non-relevant articles about herbal therapy, or transdermal hormone therapy, the total number of relevant review articles was 177.

#### Physician attitude surveys

As physicians play a key role in medical prescription counseling, they comprise an important source of HT information for women considering menopausal symptom management. Therefore, physician’s HT attitude and practice are helpful in examining whether and how HT information is communicated and how decisions for HT usage or non-usage are made.

Using Medline, we searched for surveys of physician attitude and behavior for the period post WHI to the end of 2009 and from 2012 to 2014 using Boolean operators and the terms (“attitude” OR “perception,”) AND “physician” AND “survey” AND (“hormone replacement therapy” OR “menopausal hormone therapy”). Initial results totaled 319 articles. After excluding non-English, pre WHI, non-U.S. based surveys, surveys of non-physician HT attitudes and commentaries, 18 articles with 19 surveys of physicians resulted, two of which were qualitative surveys.

#### Media coverage

Media are major sources of information and play a significant role in influencing women’s decision to stop using HT [49,55–56]. In order to cover a wide range of information sources, we selected major media outlets specifically television, newspapers, and magazines with websites on the basis of circulation and relevant audience demographics with the widest reach of menopausal women aged 50 to 60 years.

The television outlets comprised the three major US broadcast networks ABC, CBS and NBC. The newspapers were *New York Times*, *Wall Street Journal* and *USA Today* and the magazines were *Better Homes and Gardens*, *Ladies Home Journal* and *Oprah*. In addition, we searched the magazine websites for HT reports from the specified periods, post WHI to 2003, 2008–2009 and 2012–2014. Because news articles in the third period were few (six in all), these were integrated into the second period.

These media sources were chosen because of their large circulation or viewership. According to the Alliance for Audited Media (formerly the Audit Bureau of Circulations), an independent US organization providing print media and website circulation audits, the top three circulated daily newspapers are *Wall Street Journal* (2,117,796), *USA Today* (1,829,099), and *New York Times* (916,911) [57]. The Nielsen Company, the largest US audience measurement organization, reports that NBC, ABC, and CBS produce the top three national network news programs for the 2009–2010 broadcast season—NBC averages 9,131,000 viewers/night, ABC averages 8,056,000, and CBS averages 5,991,000 viewers [58]. For magazine selection, besides overall circulation, MRI+ database was used to determine whether the magazine’s primary readership corresponds with the target age group of women (59), based on their annual survey, an index score of more than 100 (which represents the average) means that the readership among the target group was above average, and *Better Homes and Gardens*, *Ladies Home Journal* and *Oprah* all had above average index scores for the target group (136, 143, 116 respectively) [59].

HT-focused media reports published during 2002–03, 2008–09 and 2012–14 were tracked through Lexis-Nexis and ProQuest Research Libraries using key words, “hormone replacement therapy,” “hormone therapy,” and “hormone therapy side-effects.” A total of 96 transcripts (45 in CBS, 15 in ABC and 35 in NBC) was identified for 2002–2003, and 30 transcripts (6 in CBS, 7 in ABC, and 16 in NBC) were located for 2008–2009. Only one (NBC) was located in 2012–14. In the 2002–2003 period, one report from CBS was repeated three times on different newscasts, one report was repeated twice, and four reports were rebroadcast once. All repeats were included in the coding because they increased the exposure, albeit of the same message.

The newspaper search returned a total of 134 articles in 2002–03 (74 in *The New York Times*, 29 in *USA Today* and 31 from *Wall Street Journal*) and 41 articles in 2008–09 (15 in *New York Times*, 8 in *USA Today*, 18 in *Wall Street Journal*). In 2012–14, five articles were located (3 in *New York Times* and two in *USA Today*).

Magazine HT articles totaled 10 articles in the 2002–2003 period (1 from *Better Homes and Gardens*, 5 from *Ladies Home Journal*, and 4 from *Oprah)*. For the 2008–2009 period, the search yielded 41 articles (19 from *Better Homes and Gardens*’ website, 9 came from *Ladies Home Journal*, and 13 from Oprah’s *O Magazine* and its website which includes TV and webcast transcripts). In 2012–14, no magazine stories were found.

### Coding scheme

The coding schemes for the HT review journal articles and media reports are similar in coding for valence. This was coded as: 1) benefits outweigh risks, 2) risks outweigh benefits, or 3) the article includes both benefits and risks and does not take a position, and/or suggests consulting with physicians, in this case, the article will be coded as balanced, because such a suggestion warrants further investigation by the readers. Specifically, the HT risks included breast cancer, cardiovascular risks/strokes, and other risks e.g., dementia. Description of HT benefits included on-label items (relief of menopausal symptoms such as hot flashes, night sweats, vaginal dryness and osteoporosis prevention) and off-label items (colon cancer prevention, heart disease prevention, better skin, increased libido, or improvement of cognitive function). The length of description as well as the location in which they appear will be taken into consideration (e.g., the headline attracts more attention than the middle paragraph). Additionally, the medical journal abstracts were coded for the primary health issue discussed (e.g., the main theme of the article) and the authors’ country of affiliation.

The physician attitude surveys were qualitatively content-analyzed to assess US physicians’ HT orientation after WHI’s publication by extracting key findings focused on attitudes toward HT, HT prescribing practices and trends in physicians HT recommendations. Basic themes included, 1) prescription practice (degree of change after WHI, prescription duration, and dosage level), 2) attitude towards WHI findings (degree of support), and 3) factors that influence HT prescription (e.g., patient’s symptom severity and risk factors).

We noted the percentages affiliated with the principal WHI findings in order to identify whether a clear majority was present or responses were varied. We differentiated between the medical specialties when these were reported such as obstetrician-gynecologists (OBGYN), internists, primary care physicians (PCP), family physicians, and others. Finally, we coded the overall sample of physicians in each of the studies as being generally positive, negative or varied in their attitude towards HT.

A “positive” code was assigned when most or a majority of the sample was more likely to favor HT e.g., when 97% think patients will receive positive HT benefits or 90% indicate that benefits outweigh risks.

A “negative” code was assigned when most or a majority of the physicians was more likely to be negative towards HT e.g., when most physicians were not in favor of using HT.

A “varied” code was assigned when the physicians have a range of attitudes or variable opinions reported e.g., when 66% OBGYN vs 35% internists favor HT or when 30% felt that HT use would prolong women’s lives while 36% felt it would not and 33% were uncertain.

Two independent coders coded a sample of 10 percent of the journal abstracts and news reports for intercoder reliability. The results showed a *kappa* of 0.76 which is considered substantial agreement [60,61]. No intercoder reliability was reported for the physician attitude surveys as these were qualitatively analyzed. The medical review journals articles and news media stories were searched for and accessed from November 2010 to April 2011 and from October to December 2015.

Where appropriate, the chi-square test or the Fisher exact test was applied and probability levels of significance set at p < .05. SPSS software was used for statistical analyses. The data are available in two supplemental files.

## Results

Q1: Post WHI, what are medical review articles’ HT orientation and how will they contribute to a state-of-the-science gap?

A mixed orientation was found. The valence of the 177 review articles was mixed (*Chi-Square* = 5.32, *df* = 2, *p*<ns) with approximately similar proportions of articles focused on benefits (40.1%), risks (26.0%) and both benefits and risks (33.9%). This mixed orientation leads to ambiguity among physicians intent on utilizing the most up-to-date information in their medical practice. See S1 Table.

Q2: Post WHI, what are physicians’ HT attitudes and how will they contribute to the state-of-the-science?

In the 19 physician HT attitude surveys analyzed, a combined total of 9864 physicians comprising 5389 (60.1%) obstetricians-gynecologists, 2230 (24.9%) physicians, 716 (8%) family physicians and 629 (7%) internists was queried. Overall, more than half or 10 physician surveys showed a positive HT attitude, eight reflected a varied attitude towards HT and one did not support HT use. Therefore, physician surveys seemed to reflect a positive leaning towards HT and also a varied one and contributed to a state-of-the-science gap. Table 1.

**Table 1:** Physician and HT survey characteristics and main findings.

Article	Survey Date	Affiliation/ Funding	Sample	Main Findings
**Positive HT Attitude**				
Birkhauser MH, Reineck I. Current trends in hormone replacement therapy: perceptions and usage. Climacteric 2008; 11(3):192–200.	June-Aug 2007	Funding from Novo Nordisk FemCare AG, Wyeth, Schering, Solvay Pharmaceuticals	600 physicians: 150 in the US	97% believed their patients received positive benefits from HT. 90% believed HT benefits outweighed the risks in suitable patients. 78% believed the negative HT media reports to be unjustified.
Burg MA, Fraser K, Gui S, Grant K, Kosch SG, Nierenberg B, et al. Treatment of Menopausal Symptoms in Family Medicine Settings following the Women's Health Initiative Findings. J Am Board Fam Med 2006; 19(2):122–131.	2004		210: 62 faculty, 148 residents, Florida	79% changed their HT practice after WHI (85.8% female vs 71.6% male). 69.1% prescribed HT for vaginal dryness, 50.9% for vasomotor symptoms, 50.9% for irregular menses and 39.2% for decreased libido.
Morgan MA, Lawrence H 3rd, Schulkin J. Obstetrician-gynecologists' approach to well-woman care. Obstet Gynecol 2010; 116(3):715–722.	May-Oct 2009	Authors from ACOG; grant from the Maternal and Child Health Bureau	513 OBGYN	73% would prescribe HT when patients are interested in HT and 69% would prescribe it for menopause.
Newton KM, Reed SD, Grothaus LC, La Croix AZ, Nekhlyudov L, Ehrlich K, et al. Hormone therapy discontinuation: physician practices after the Women's Health Initiative. Menopause 2010; 17(4), 734–740.	Dec 2005-May 2006	Funding from National Institutes of Health through National Institute of Aging Grant	483: 60 OBGYN, 423 PCP	91% would recommend tapering HT usage with 26% of this group decreasing dosage, 10% decreasing frequency of use, 60% decreasing both dosage and frequency of use and 8% recommending immediate cessation. 40% perceived that most women wanted to discontinue HT use. 24% of the family physicians encouraged women to stop HT as soon as possible while 8% of OBGYN did so.
Power ML, Zinberg S, Schulkin J. A survey of obstetrician-gynecologists concerning practice patterns and attitudes toward hormone therapy. Menopause 2006; 13(3), 434–441.	Nov 2003	Research dept. of ACOG	644 OBGYN	97.2% believed HT to be a viable treatment for hot flashes and 93.5% would recommend it for vaginal atrophy. 49.1% were unconvinced by the WHI results and 48.1% disagreed with the decision to terminate the trial. 39.3% believed that HT benefits outweighed the risks for a majority of postmenopausal women. OBGYN who recently completed their residency were more likely to accept the trial results.
Power Ml., Schulkin J, Rossouw J. Evolving practice patterns and attitudes toward hormone therapy of obstetrician-gynecologists. Menopause 2007; 14(1), 20.	Dec 2004-Mar 2005	Research dept. of ACOG	902 OBGYN	OBGYN continued to remain skeptical about the WHI results. 47.7% did not find the WHI results convincing and 33.2% did not agree with the decision to end the trial.
Power ML, Baron J, Schulkin J. Factors associated with obstetrician-gynecologists' response to the Women's Health Initiative trial of combined hormone therapy. Med Decis Making 2008; 28(3), 411–418.	Nov 2003	Research dept. of ACOG	703 OBGYN	79.7% prescribed HT to more than half of their patients but only 25.9% did so the past 6 months. 62.7% reported that they were unlikely to change their practice. 23.9% would prescribe HT only when patients asked for it. Female OBGYN (37.3%) found the WHI results more convincing than their male counterparts (22.6%). Overall, 9.1% were unconvinced by the EPT WHI results and 46.9% disagreed with the decision to stop the EPT trial.
Rolnick SJ, Jackson J, Kopher R, Defor TA. Provider management of menopause after the findings of the Women's Health Initiative. Menopause 2007; 14(3), 441–449.	Published 2007, no survey date reported	Funding from HealthPartners Research Foundation and Merck and Co. Inc.	200 medical providers from Midwestern Health Organization	89% prescribed HT for menopausal symptom relief, 74% reported prescribing lower doses and 73% encouraged women to use HT for shorter periods.
Sangi-Haghpeykar H, Poindexter AN 3rd. Physicians' views and practices concerning menopausal hormone therapy. Maturitas 2007; 56(1), 30–37.	2003	Funded in part by Wyeth	1614: 633 OBGYN, 571 family physicians, 410 internists	Physicians seemed to accept short-term use of EPT. 62% believed HT would relieve menopausal symptoms for the short term assuming no contraindications: 82% OBGYN, 54% family physicians and 42% internists.
Sievert LL, Saliba M, Reher D, Sahel A, Hoyer D, Deeb M, et al. The medical management of menopause: a four-country comparison care in urban areas. Maturitas 2008; 59(1), 7–21.	Feb-Oct 2002		59 physicians from Worcester, MA; 210 physicians from 3 other countries	54% of US physicians believed symptom relief to be a large HT benefit and 41% were seriously concerned about breast cancer risks. 10% rated women as well informed about HT knowledge
**Negative HT Attitude**				
Nekhlyudov L, Bush T, Bonomi AE, Ludman EJ, Newton KM. Physicians' and Women's Views on Hormone Therapy and Breast Cancer Risk After the WHI: A Qualitative Study. Women & Health, 2009; 49(4), 280–293.	Jan-Dec 2005		22 physicians: 10 WA, 10 MA	Most physicians did not favor HT particularly for women with breast cancer risk factors and for long term use. Menopausal symptoms were often the indication to prescribe HT although some physicians were more reluctant than others to do so. The WHI findings changed their HT beliefs although some remained skeptical.
**Varied HT Attitude**				
Brett AS, Carney PI, McKeown RE. Brief report: attitudes toward hormone therapy after the Women's Health Initiative: a comparison of internists and gynecologists. J Gen Intern Med 2005; 20(5):416–418.	Oct 2002	Grant from the Palmetto Health Alliance, Columbia, SC	576 physicians: 357 GYN, 219 internists	More GYN (66%) than internists (35%) have a permissible attitude towards continuing HT.
Bush TM, Bonomi AE, Nekhlyudov L, Ludman EJ, Reed SD, Connelly MT, et al. How the Women's Health Initiative (WHI) influenced physicians' practice and attitudes. J Gen Intern Med 2007; 22(9):1311–1316.	After WHI		22 physicians (family physicians, internists, GYN): 10 WA, 10 MA	WHI was a groundbreaking study and influenced how physicians counsel women. Physicians varied in their opinions about HT and the scientific evidence. They used various discontinuation strategies and reported that they lacked information and needed decision aids about menopause and HT.
Devi G, Sugiguchi F, Pedersen AT, Abrassart D, Glodowski M, Nachtigall L. Current attitudes on self-use and prescription of hormone therapy among New York City gynaecologists. Menopause Int 2013; 19(3):121–126.	Published 2013. No survey date reported.		209 NYC OBGYN	They agreed with the WHI findings. 74% of female GYN and female partners of male GYN use/have used HT. 27.3% of male GYN and 12.3% of female GYN recommended HT to all menopausal women regardless of contraindications. They remained divided in their HT attitude: 30% felt HT prolonged women's lives, 36% felt HT was not useful in prolonging women's lives and 33% were unsure.
Lakey SL, Reed SD, LaCroix AZ, Grothaus L, Newton KM. Self-Reported Changes in Providers' Hormone Therapy Prescribing and Counseling Practices After the Women's Health Initiative. Journal of Women's Health 2010; 19(12):2175–2181.	Dec 2005-May 2006		592: 79 OBGYN, 513 internists	OBGYN and internists differed in HT prescribing and counselling: OBGYN were more likely to believe that balanced HT benefits outweighed the risks: 37.2% vs 19.2% PCPs. They were also more likely to continue to recommend HT to women: 59% OBGYN vs 22.9% PCP.
Power ML, Anderson BL, Schulkin J. Attitudes of obstetrician-gynecologists toward the evidence from the Women's Health Initiative hormone therapy trials remain generally skeptical. Menopause 2009; 16(3), 500–508.	I: Sep 2005-Jan 2006, II: Dec 2006-Jan 2007	Research dept. of ACOG	Study I: 800 OBGYN, Study II: 286 OBGYN from Collaborative Ambulatory Research Network CARN	In general, OBGYN remained skeptical of the WHI findings. Study I: 47.7% did not find the WHI results convincing: 54.2% male vs 39.8% female. 72.9% would not change their prescribing practice with 11.4% prescribing HT only upon patients' request. Study II: 59.9% did not find the WHI results convincing and 48.8% disagreed with the decision to end the trial. 23.9% would prescribe HT only upon patients' request.
Spangler L, Reed SD, Nekhyludov L, Grothaus LC, LaCroix AZ, Newton KM. Provider attributes associated with hormone therapy prescribing frequency. Menopause 2009; 16(4), 810–816.	Dec 2005	Funding from National Institutes of Health	379 physicians from NE and NW United States	58% were less likely to recommend HT for menopausal symptoms and 41.9% encouraged discontinuation of HT soon. Overall, OBGYN wrote more prescriptions per visit than PCPs and in the NW, OBGYN prescribed HT more frequently compared to PCPs.
Williams RS, Christie D, Sistrom C. Assessment of the understanding of the risks and benefits of hormone replacement therapy (HRT) in primary care physicians. Am J Obstet Gynecol 2004; 193(2), 551–556; discussion 556–558.	Mar 2004		600 Florida physicians: 203 OBGYN, 145 family physicians, 219 internists, 33 others	Overall, physicians would prescribe HT for 3 to 5 years: 43% OBGYN, 34% family physicians, 21% internists and 33% others. 28% of the physicians correctly assessed the HT risks and benefits in the WHI results. OBGYN rated HT more favorable compared to internists (3.8 vs 2.3 rating on a 1 to 5 scale).

The results also showed clear attitude differences between gynecologists and primary care physicians which included internists and family physicians. Overall, the physicians specializing in women’s medical conditions were more willing to prescribe hormone therapy to their patients than internists or family physicians.

To elaborate, a study found that 50% of the gynecologists had a favorable attitude towards hormone therapy compared to 29% of the internists [62]. A majority of 600 physicians (90 to 97%) believed that HT benefits outweighed the risks and that their patients received positive benefits from HT [63] and another majority (69% to 97%) of 513 and 703 surveyed OBGYNs would prescribe HT for menopause [64].

Five studies sponsored by the American College of Gynecology published between 2006 and 2009 showed that half of the profession was not persuaded by the WHI results [65–68]. A 2005–06 survey among 483 physicians found that 91% advised HT tapering while 8% suggested immediate HT cessation [69]. Among 209 gynecologists, 30% thought HT use prolonged women’s lives while 36% did not, and 33% were uncertain [70]. As a consequence, the continued support of HT use among physicians as well as the varied attitudes among the different medical specialties contributed to a state-of-the-science gap.

H1A: Post WHI, media coverage of HT by television news media will reflect coverage consistent with the state-of-the-science that HT risks outweigh the benefits.

This was true in the later period but not in the immediate post WHI period in 2003 when the emphasis was on both risks and benefits (*Chi-Square* = 14.9, *df* = 2, *p* < .001). Table 2. See S2 Table.

**Table 2:** Hormone therapy TV news coverage by valence.

Valence	WHI 2002–03	Post WHI 2008–09, 2012–14
	N (%)	N (%)
Benefits>risks	3 (3.1)	3 (9.7)
Risks>benefits	25 (26.0)	18 (58.1)
Risks & benefits	68 (70.9)	10 (32.3)
Total	96 (100)	31 (100)

H1B: Post WHI, media coverage of HT by newspaper outlets will reflect coverage consistent with the state-of-the-science that HT risks outweigh the benefits.

Newspaper HT coverage focused primarily on both risks and benefits in both 2003 and the later period although the risk coverage increased substantially between the periods. H1B was not supported (*Chi-Square* = 5.35, *df* = 2, *p*<ns) as newspapers predominantly covered both sides of the issue. Overall story frequency declined substantially from 2002–03 to the later period. Since coverage in both periods focused more on both risks and benefits, newspaper stories contributed to a state-of-the-science gap. Table 3.

**Table 3:** Hormone therapy newspaper coverage by valence.

Valence	WHI 2002–03	Post WHI 2008–09, 2012–14
	N (%)	N (%)
Benefits>risks	13 (9.7)	1 (2.2)
Risks>benefits	41 (30.6)	21 (46.7)
Risks & benefits	80 (59.7)	23 (51.1)
Total	134 (100)	45 (100)

H1C: Post WHI, in line with providing service information, magazines and their internet coverage of HT will favor HT use and comprise a state-of-the-science gap.

H1C was partially supported. This was true in the later period but not in the immediate post WHI 2002–03 period when the emphasis was on both risks and benefits (*Chi-Square* = 8.2, *df* = 2, *p* < .05). Table 4.

**Table 4:** Hormone therapy magazine coverage by valence.

Valence	WHI 2002–03	Post WHI 2008–09, 2012–14
	N (%)	N (%)
Benefits>risks	0 (0.0)	20 (43.8)
Risks>benefits	3 (30.0)	5 (12.2)
Risks & benefits	7 (70.0)	16 (39.0)
Total	10 (100)	41 (100)

## Discussion

The decline in standard HT dose usage after 2002 clearly reflected the state-of-the-science which underscored increased risks of HT use while the increase in low dose HT prescriptions demonstrated a state-of-the-science gap because of the absence of conclusive evidence about the risks and benefits of low dose HT use. Physicians are the primary gatekeepers of prescriptions, therefore the increase in low dose HT prescriptions must be attributed to positive prevailing medical attitudes and beliefs about HT use in low doses among these medical professionals.

The present study found a state-of-the-science gap among medical journal reviews of HT use and our analysis showed that overall, the reviews have a mixed orientation towards hormone therapy with approximately equal proportions emphasizing the benefits, risks and both benefits and risks. This study examined randomly selected HT reviews for the entire period 2002 to 2014 and the results nonetheless suggest the disparity between the WHI findings and medical journal articles did not disappear over time. It is proposed that the mixed messages may be the result of successful medical marketing strategy by Wyeth through its proxy medical education and communication company to emphasize the benefits of HT and recommend its usage. For the most part, doctors rely on peer-reviewed scientific medical journals to keep up-to-date with their medical practice. Without a clear trend of studies in the medical literature reinforcing the conclusions of the WHI landmark study– 1) HT use, specifically estrogen-progestin, leads to increased risks of breast cancer, heart attacks, strokes and blood clots, and 2) using estrogen alone in women with a prior hysterectomy had more balanced risks and benefits for women 50–59 including decreased breast cancer but increased strokes and blood clots—it is not surprising that our study’s assessment of physician surveys, particularly among gynecologists and specialists in women’s health, revealed a pro-HT orientation among gynecologists. HT does relieve menopausal symptoms but increases other health risks. In the face of patients in distress, physicians seem ready to prescribe therapies that will help to bring relief.

Studies have pointed to physicians as the most trusted sources of medical information and patients, more often than not, are likely to comply with their physicians’ advice whether it is to use or not use HT. Therefore, in the face of mixed HT reviews, it is no surprise that the views of obstetricians/gynecologists and primary care physicians varied and will continue to vary depending on their medical specialty or subspecialty. The differing advice that these physicians provide to their patients contributes to the state-of-the-science gap in HT usage.

On the media front, the different HT coverage by the different media revealed that different media platforms may have different functions. News media report the news. The Women’s Health Initiative was major headline news in 2002 hence TV news media coverage showed that there were many more reports about HT then with the majority of stories focused on both benefits and risks. At that time, TV coverage reflected a state-of-the-science gap. In the later period 2008–09 and 2012–14, HT was no longer headline news therefore the reports are fewer, but this time HT was more often cited as a risk factor. TV news reinforced the state-of-the-science.

By the same token, newspapers also report the news. Unlike its electronic cousin, HT newspaper coverage more likely included both sides and focused on both risk and benefits. Covering both sides may preempt charges of bias and assuming an advocacy position which journalists eschew [71]. However, in this case it contributed to the state-of-the-science gap. In order to better assist their news consumers make better healthcare decisions, health and science journalists may wish to reevaluate their professional reporting principles and focus primarily on the “science” resulting from medical research which does not have “both sides.” Recognizing the rigor of the scientific methodology behind scientific evidence, findings, reports and reviews would help in differentiating the science from the “noise.”

Women’s magazines provide advice and tips to women. Just after the WHI study in 2003, magazines were more likely to report both the benefits and risks of HT just as newspapers did. When HT use was the norm for the past four decades, it was difficult to change the paradigm and magazines contributed to a state-of-the-science gap in HT use.

With the objective of providing service information in 2009, magazines and their corresponding internet sites were found to promote the benefits of HT use in spite of the WHI finding that HT use is associated with serious health risks. In the later period, women were still looking for advice about HT use, therefore magazines stepped in to offer guidelines. HT coverage increased from the previous period in order to provide more information. In the case of women’s magazines, they provide advice and tips to women on HT use. For example, an entire issue of Oprah’s *O* magazine and an episode of her talk show focused on menopause and HT concerns. Magazine coverage analyzed at this time also tended not to mention breast cancer risks and cardiovascular risks and consequently, they continued to contribute to the state-of-the-science gap in HT use.

In summary, we have seen that medical journal review articles, physicians, and media reports all provided varying view points and valences towards hormone therapy use without any unifying underpinning. The flow of medical information seems to originate and diffuse from the scientific medical journals to the medical professionals and specialists, and from these journals and practitioners to media reports, as well as to the public. Therefore, it is not surprising that peri- and menopausal women end up having a limited knowledge about the actual risks and benefits of hormone therapy and low dose hormone therapy increased from 2003 to 2009. We have a state-of-the-science gap in HT use.

The current advisory about HT by the US Preventive Services Task Force (USPSTF) applies to postmenopausal women and is against the use of combined estrogen and progestin for the prevention of chronic medical conditions, and against estrogen use likewise among postmenopausal women with a hysterectomy [8]. The risks outweigh the benefits [8]. Estrogen plus progestin, and estrogen alone, both “decreased risk for fractures but increased risk for stroke, thromboembolic events, gallbladder disease, and urinary incontinence. Estrogen plus progestin increased risk for breast cancer and probable dementia, whereas estrogen alone decreased risk for breast cancer.” [8] Finally, this recommendation also does not apply to women under 50 years who have had surgical menopause [8]. The USPSTF comprises a panel of 14 national experts appointed by the director of the US Agency of HealthCare Research and Quality (AHRQ) to “improve the health of all Americans by making evidence-based recommendations about clinical preventive services such as screenings, counseling services, and preventive medications.” [8]. Its recommendation would reflect the state-of-the science.

In a 2012 Securities and Exchange filing, Pfizer Inc. reported paying $896 million to resolve about 60% of the cases that alleged its drugs to treat menopausal symptoms in women caused cancer [72,73]. By this time, the company had settled 6,000 lawsuits claiming Prempro and other hormone-replacement drugs caused breast cancer and it had also set aside $330 million to resolve the remaining 4,000 lawsuits for a total of $1.2 billion [72,73]. Court documents have profiled how the drug manufacturer targeted influential doctors and professional medical societies with positive messages about hormone replacement therapy, used celebrity advertising, medical education courses and the authoring of scientific publications to likewise promote its product [14]. Will this same scenario play out with the low dose hormone therapy for which the evidence of benefits and risks are yet unknown? Time will tell. Ultimately, the public needs to be made fully aware of all the risks associated with various therapies including HT use so that they can make informed decisions about their health. Consistently ensuring the disclosure of journal authors’ financial interests in and associations with pharmaceutical companies or any other private enterprise would be a key step.

This study examined HT news data for three time periods, 2002–03, 2008–09 and 2012–14. Further research would be more likely to establish a longitudinal trend if the data-set covers the entire 2002–2014 period. We analyzed three media platforms and selected three outlets per medium. More outlets may strengthen study reliability. Other media such as the internet merits analysis. Finally, the study focused primarily on US media and physicians without exploring the state-of-the-science gap in other countries.

Future research should consider examining the state-of-the-science gap in regard to other medical conditions. Other and more factors that contribute to shaping the gap in HT usage should be considered including the off-label benefits of HT such as youthful appearance. Another methodological procedure would be to survey newly menopausal and postmenopausal women at the patient level to ascertain their HT knowledge, attitudes and behavior/usage as well as their sources of HT information and corresponding level of trust and reliability. Likewise, understanding how gynecologists and physicians keep up to date with medical reports and make decisions about HT recommendations merits study. Surveying international perspectives would enhance our understanding of the state-of-the-science gap. Finally, our study qualitatively content analyzed physician HT attitudes post WHI. A quantitative systematic review awaits investigation.

## Supporting information

S1 Table Hormone therapy medical review data. (ZIP)

S2 Table Hormone therapy news media data. (ZIP)
